# Analysis of Influencing Factors for Choosing Dermatology as a Future Career: A Cross-Sectional Study Among Medical Students at King Abdulaziz University

**DOI:** 10.7759/cureus.66021

**Published:** 2024-08-02

**Authors:** Albatool M Balkhair, Maan M Almaghrabi, Hanin M Banjer, Roba Waznah, Malika Almadani, Jehad Hariri

**Affiliations:** 1 Dermatology, King Abdulaziz University Hospital, Jeddah, SAU

**Keywords:** influencing factors, saudi arabia, career, medical students, dermatology

## Abstract

Background: Determining the influencing factors of choice of medical specialty is key to a balanced distribution of physicians across specialties. Dermatology, the specialty concerned with treating skin disorders, is known for being among the most wanted; however, studies identifying the factors that attract students to this specialty are lacking. Our study aimed to investigate the influencing factors of the choice of dermatology as a career in clinical-year medical students.

Methods: We conducted a cross-sectional study with a sample of clinical-year medical students from King Abdulaziz University, Jeddah, Saudi Arabia, between 2020 and 2021. We collected data using an online self-administered questionnaire; we replicated a questionnaire present in prior research. We compared categorical data using the chi-square and Fisher’s exact tests.

Results: In total, there were 252 participants, with 30 (11.9%) choosing dermatology as a specialty. Over half showed an average grade of more than 4.5 (66.7%), and 83.3% were female. The significant influencing factors of students’ choice of dermatology as a career were: the likelihood of dermatologists influencing patients’ lives (p=0.000), opportunities to conduct research in dermatology (P=0.000), how Dermatology allows people to have a satisfying family life (P=0.001), and opportunities for part-time work in dermatology (p=0.000).

Conclusion: Many factors influenced the choice of a future medical specialty in our sample. Focusing on these factors while guiding students to choose their specialty may enable a promising next generation of physicians.

## Introduction

The selection of a medical specialty could be an overwhelming challenge for both students and interns; concomitantly, it is a significant indicator of the future availability of physicians across specialties for a region. Research shows that recognizing the variables driving specialty preferences can enhance our understanding of students’ interests for specific specialties and allow career advisors to offer helpful guidance for medical students [1,2]. On the topic, dermatology, which is the medical specialty concerned with the diagnosis, recovery, and prevention of skin and subcutaneous tissue diseases [3], has often been regarded as one of the most competitive specialties in Saudi Arabia and worldwide [4,5].

In Saudi Arabia, the dermatology residency training program is collaborative, in that trainees spend a fixed duration (four years) practicing the procedures related to dermatology and its subspecialties in the hospital that has the respective subspecialty or a dermatology department. During the program, there are yearly examinations that are conducted to promote residents; applicants who pass the final board examination enter the Saudi Board of Dermatology [6,7].

There are many factors influencing medical students’ choice of future specialty, and these include a good lifestyle and a satisfying family life. Additionally, students’ personalities and habits directly impact their medical specialty choice [1,8]. On this topic, various studies show that the characteristic of the dermatology specialty to grant its practitioners a controllable lifestyle (CL) is a potent factor that influences students’ choice of specialty [9]; these studies also show that every year, an increasing number of medical students are attracted to the dermatology specialty.

There are various studies on the factors influencing medical students’ choice of dermatology as their future specialty. A questionnaire-based study from 2010, conducted with 644 Australian medical students, showed that dermatology ranked first on the topic of lifestyle friendliness [10]. Furthermore, in 2018, some researchers used multipurpose, longitudinal surveys to analyze the careers of doctors trained in the United Kingdom who graduated between 1974 and 2015, attempting to determine their career choices for dermatology and the factors influencing their career progression [11]. With an initial sample of 41,412 participants, they showed that the highest certainty among the doctors regarding the choice of specialty was for dermatology; further, doctors’ commitment to dermatology increased from 18% in the first year to 72.6% in the fifth year. Moreover, hours/working conditions were a major influencing factor for choosing dermatology as a career, followed by commitment and enthusiasm [11]. In 2009, another group of researchers conducted a cross-sectional study in the United Kingdom exploring career choices in dermatology; they discovered that the most compelling factor for doctors to consider a career in dermatology is contact with an influential dermatologist [12].

Locally, in Dammam, Saudi Arabia, a cross-sectional study conducted in 2014 showed that lifestyle was the most influential factor for both medical students and interns in determining their future specialty [13]. In Jeddah, Saudi Arabia, a cross-sectional study conducted in 2020 study at King Saud bin Abdulaziz University for Health Science (KSAU-HS) demonstrated the perceptions and attitudes of medical students toward dermatology as a future specialty. Of the 121 participants, nearly 6.6% chose dermatology as a future career, and the major influencing factor was the interest in being a dermatologist [14]. In Riyadh, Saudi Arabia, a cross-sectional study conducted in 2016 measured the reasons for choosing dermatology as a career at KSAU-HS; with a sample of 118 medical students, it showed that the appeal of being a dermatologist, dermatologists’ satisfaction with family life, reliance on clinical diagnostic skills, and research opportunities in dermatology were the most important factors influencing the choice of dermatology as a specialty [5].

Although dermatology is one of the most competitive specialties and there is various research on the topic, we observed that no studies are exploring the factors influencing students’ choice of dermatology as a specialty at King Abdulaziz University, Jeddah, Saudi Arabia. In addition, these aforementioned studies had a frequent limitation: small sample sizes. Therefore, we aimed to investigate the factors influencing the choice of dermatology as a career among clinical-year medical students during the academic year of 2020-2021.

## Materials and methods

Study design and ethical information

This cross-sectional study was approved by the institutional review board (reference number: 536-20), under the command of the Dermatology Department at King Abdulaziz University Hospital in Jeddah, Saudi Arabia. Informed consent was obtained from each subject before participation in this study, with full compliance with the principles of the Helsinki Declaration. It was conducted between June and July 2021.

Participants

In all, 300 medical students participated by completing an online self-administered questionnaire. The sample size was calculated using Raosoft, considering a margin of error of 5% and a 95% confidence level [15]. We used non-probability sampling for recruitment, and only medical students in their clinical years (that is, fourth-, fifth-, and sixth-year medical students) were allowed to participate. Visiting medical students were excluded. We stressed for all participants that participation in this research was completely voluntary and that they could withdraw from the study at any time without the need for a justification or incurring any penalty. Participants were not required to provide their identities for completing the survey, but they were requested to provide informed consent prior to being able to complete the questionnaire.

Procedures and questionnaire

We used a validated self-administered English online questionnaire that was developed by authors of prior similar research conducted at KSAU-HS [5,14]; our use of this questionnaire was approved by the original authors. We used Google Forms for questionnaire application and data collection. The first part of the questionnaire contained questions about participants’ sociodemographic characteristics, including age, marital status, academic year, and grade point average (GPA). The second part of the questionnaire comprised 16 items, and they were responded to on a three-item scale, ranging from less attractive, more attractive, or no influence regarding factors that affected their choice on choosing Dermatology as a career.

Data analysis

We used Excel (version 16.0, Microsoft Corporation, Redmond, Washington, USA) to collect and organize data, and SPSS (version 21, IBM Corp., Armonk, New York, USA) for data analysis. We used frequency and percentage to describe categorical variables, and mean and standard deviation to describe continuous variables. For comparing data, we used the chi-square test and Fisher’s exact test, with statistical significance set at a P<0.05.

## Results

In total, 252 clinical-year medical students participated, with a mean age of 22.687 ± 1.0753. Of these, most respondents were single (95%), 73 (29%) were fourth-, 84 (33.3%) were fifth-, and 95 (37.7%) were sixth-year students. Regarding GPA, nearly half of the participants had a GPA greater than 4.49 (out of 5), 38.1% scored between 4 and 4.49, and the remaining had scored less than 4. Most students chose their specialty during the clinical years: 187 (74.2%), 22 (8.7%) chose it during the basic years, and 43 (17.1%) chose it before medical school.

Further, 30 (11.9%) students chose dermatology as their preferred medical specialty. Of them, 53.3% made this choice during clinical years, while the other half made this choice between either their medical or surgical specialties. Among the 30 students who preferred dermatology, 25 were female, 14 were in their fourth year, and more than half had a GPA above 4.49. Participants’ sociodemographic characteristics are shown in Table 1.

**Table 1:** Sociodemographic data. GPA: grade point average.

Variable	Category	N (%)			P-value
		Dermatology	Other	Total	
Gender	Male	5 (16.7%)	84 (37.8%)	89 (35.3%)	0.038
	Female	25 (83%)	138 (62.2%)	163 (64.7%)	
Academic level	Fourth year	14 (46.7%)	59 (26.6%)	73 (29%)	0.073
	Fifth year	8 (26.7%)	76 (34.2%)	84 (33.3%)	
	Sixth year	8 (26.7%)	87 (39.2%)	95 (37.7%)	
Marital status	Single	26 (86.7%)	215 (96.8%)	241 (95%)	0.030
	Married	4 (13.3%)	7 (3.2%)	11 (4.4%)	
GPA/5	>4.49	20 (66.7%)	105 (47.3%)	125 (49.6%)	0.031
	4-4.49	7 (23.3%)	89 (40.1%)	96 (38.1%)	
	<4	3 (10%)	28 (12.6%)	31 (12.3%)	
When did you make your specialty choice	During clinical years	16 (53.3%)	171 (77%)	187 (74.2%)	0.012
	During basic years	6 (20%)	16 (7.2%)	22 (8.7%)	
	Before medical school	8 (26.7%)	35 (15.8%)	43 (17.1%)	

Using chi-square tests, we found that the factors that significantly influenced the choice of dermatology as a career included how dermatology allows people to have a satisfying family life (P=0.001), the opportunity to perform different dermatological procedures (P=0.004), the appeal of being a dermatologist (P=0.000), the likelihood of dermatologists to influence patients’ lives (P=0.000), opportunities to conduct research in dermatology (P=0.000), reliance on clinical diagnostic skills (P=0.001), portrayal of different specialties in the media (P=0.004), opportunities for part-time work in dermatology (p=0.000, and the variety of patients (all ages, both genders); p=0.042), as detailed in Table 2. Further, high income and degree of stress did not significantly influence the choice of dermatology as a career.

**Table 2:** Reasons for choosing dermatology as a future specialty.

Question	Answer	Specialty N (%)		p-value
		Dermatology	Other	
The free time away from work	Less attractive	1 (3.3%)	32 (14.4%)	0.193
	No influence	7 (23.3%)	56 (25.2%)	
	More attractive	22 (73.3%)	134 (60.4%)	
The appeal of being a dermatologist	Less attractive	1 (3.3%)	88 (39.6%)	0.000
	No influence	14 (46.7%)	109 (49.1%)	
	More attractive	15 (50%)	25 (11.3%)	
How dermatologists role model a satisfying family life	Less attractive	2 (6.7%)	51 (23%)	0.001
	No influence	8 (26.7%)	98 (44.1%)	
	More attractive	20 (66.7%)	73 (32.9%)	
The difficulty of getting into the dermatology residency program	Less attractive	14 (46.7%)	102 (45.9%)	0.282
	No influence	5 (16.7%)	63 (28.4%)	
	More attractive	11 (36.7%)	57 (25.7%)	
Opportunities for part-time work in dermatology	Less attractive	2 (6.7%)	50 (22.5%)	0.000
	No influence	6 (20%)	99 (44.6%)	
	More attractive	22 (73.3%)	73 (32.9%)	
The variety of patients (all ages, both genders)	Less attractive	2 (6.7%)	55 (24.8%)	0.042
	No influence	16 (53.3%)	113 (50.9%)	
	More attractive	12 (40%)	54 (24.3%)	
The length of residency years	Less attractive	5 (16.7%)	55 (24.8%)	0.054
	No influence	12 (40%)	116 (52.3%)	
	More attractive	13 (43.3%)	51 (23%)	
The opportunity to perform different dermatological procedures	Less attractive	3 (10%)	49 (22.1%)	0.004
	No influence	6 (20%)	89 (40.1%)	
	More attractive	21 (70%)	84 (37.8%)	
Reliance on clinical diagnostic skills	Less attractive	0 (0%)	55 (24.8%)	0.001
	No influence	11 (36.7%)	90 (40.5%)	
	More attractive	19 (63.3%)	77 (34.7%)	
Portrayal of different specialties in the media	Less attractive	1 (3.3%)	49 (22.1%)	0.004
	No influence	15 (50%)	123 (55.4%)	
	More attractive	14 (46.7%)	50 (22.5%)	
The likelihood of dermatologists to influence patients’ lives	Less attractive	2 (6.7%)	52 (23.4%)	0.000
	No influence	9 (30%)	116 (52.3%)	
	More attractive	19 (63.3%)	54 (24.3%)	
Opportunities to conduct research in dermatology	Less attractive	3 (10%)	79 (35.6%)	0.000
	No influence	5 (16.7%)	119 (53.6%)	
	More attractive	22 (73.3%)	24 (10.8%)	
The high income	Less attractive	2 (6.7%)	26 (11.7%)	0.230
	No influence	4 (13.3%)	56 (25.2%)	
	More attractive	24 (80%)	140 (63.1%)	
The degree of stress	Less attractive	3 (10%)	64 (28.8%)	0.067
	No influence	8 (26.7%)	58 (26.1%)	
	More attractive	19 (63.3%)	100 (45%)	
Unsatisfied patients	Less attractive	9 (30%)	99 (44.6%)	0.150
	No influence	14 (46.7%)	96 (43.2%)	
	More attractive	7 (23.3%)	27 (12.2%)	
Private sector opportunities	Less attractive	3 (10%)	29 (13.1%)	0.074
	No influence	5 (16.7%)	79 (35.6%)	
	More attractive	22 (73.3%)	114 (51.4%)	

## Discussion

In this study, we had the primary goal of assessing the factors influencing the choice of dermatology as a career among clinical-year medical students at King Abdulaziz University during the academic year of 2020-2021. We deemed this as an important investigation because the data provided by such examination may enable stakeholders, such as career advisors in medicine, to make more well-informed decisions related to advice giving for, and when assisting, medical students on their career choices. Specifically, we believe that they should focus on the most significant factors affecting students’ choice of dermatology as a career in order to help balance the demand in some specialties and to assist in the diminishment of the oversupply of doctors in others; this oversupply of some specialties and the concomitant lack of others has been well-documented in the literature [5]. In addition, we believe that future physicians should be made more aware of the reality behind the dermatology specialization, what it actually entails, and there may be a need to correct some misconceptions about this medical specialty.

Specialty preference

Our results revealed that 11.9% of the medical students preferred dermatology over other specialties. In 2016, prior research conducted in Riyadh showed that only 3% of the surveyed medical students favored dermatology over other specialties, while this number was 6.6% for a sample from Jeddah in 2020 [14,5]. Thus, our results show that there may have been an increase in Saudi students’ interest in dermatology over the years, which may owe to the rise of the popularity of cosmetic procedures [16].

Female and dermatology

In Pakistan, a study on medical specialty preference showed that most students who chose dermatology were female [17]; this finding is consonant with our findings. In Latin America, a study demonstrated that dermatology was highly associated with female students, explaining that this association could owe to the fewer hours of dedication and the number of patients involved, making it easier to balance between career and family [18]. Having a satisfying family life has also been significant. This was also shown in a study conducted to assess the work-life balance for female dermatologists in the United States, indicating that home-life and career-life balance are very relevant among female dermatologists, making the specialty very appealing for female medical students [19].

Research

Regarding dermatology research, there is a general lack of it in Saudi Arabia. This lack of studies represents a research gap, potentially motivating academicians to start researching in this field owing to the wide variety of opportunities it presents for theorists [20,21]. Indeed, the possibility of conducting research has been demonstrated to be a critical factor in choosing dermatology as a specialty [5]. Moreover, a Canadian study reported that dermatologists are an essential component of research development in the field of medicine [22]. These results are supported by our study, considering that the “opportunities to conduct research in dermatology” factor was shown to be significantly influencing students’ choice of dermatology as a career.

Influencing patients' lives

An excellent aspect of the dermatology specialty for physicians relates to the ability of the physician to affect patients’ lives. In our study, “the likelihood of dermatologists to influence patients’ lives” factor significantly influenced students’ choice of dermatology as a career; a previous study in KSAU-HS, in Jeddah, showed similar findings [14]. Indeed, skin diseases generally have a significant impact not only to one’s skin but also to one’s mental health. Several studies have associated skin diseases with feelings of anxiety, anger, and depression; therefore, it has a great impact on the patients [23,24].

Stress

Contrary to our expectations, the degree of stress related to dermatological work has not significantly influenced, in our sample, students’ choice of dermatology as a specialty. This went against our prospects because prior research, conducted in 2018, has ranked the dermatology specialty as low regarding occupational stress, indicating that there may be a lower degree of stress related to dermatology compared with other medical specialties [25].

GPA

The dermatology residency is extremely competitive and requires students to have high academic performance to be accepted, a reality that owes to the surplus of applicants and the few training positions [26]. This may explain why, in our sample, a greater number of medical students with a GPA above 4.49 preferred dermatology. This result also appeared in past research conducted in KSAU-HS, in Jeddah, where 87.5% of the students who chose dermatology as a career had a GPA greater than 4.5 [5,14].

Theoretical and practical implications

Knowing what motivates students to pursue a certain specialty is very important, especially when addressing dermatology, a specialty that is competitive worldwide. Understanding these motivations will help us recognize the key factors that influence medical students' choices. This, in turn, allows us to highlight the qualities in each specialty that medical students most care about so that, in turn, students can be content if they did not get into their first choice of specialty, which in this case, is dermatology. Furthermore, this understanding can help correct some misconceptions about the specialty and provide clarity to medical students aspiring to become future dermatologists.

Limitations

This study had several limitations. Firstly, since our study was limited to a single university in Jeddah, we could not reach our targeted sample size. Secondly, even though we only included clinical year students, however, some of the participants were never exposed to clinical dermatology rotations, and lastly some of the variables used in the questionnaire lacked clarity. Therefore, we recommend performing a multi-center study comprising different universities around Saudi Arabia.

## Conclusions

Our study aimed to identify the factors influencing clinical-year medical students’ choice of dermatology as a career in the academic year 2020-2021. In summary, several factors were found to influence this choice, such as the likelihood of dermatologists to influence patients’ lives, the appeal of being a dermatologist, the opportunity to perform different dermatological procedures, and the ability to maintain a satisfying family life. Determining medical students' preferences for specialties can aid stakeholders in the distribution of the Saudi healthcare workforce, and allowing those invested in ensuring that students tread career paths that match their life and professional requirements will be able to provide well-informed advice. Based on our results, we recommend promoting programs that help medical students select ideal specialties based on their personalities and future ambitions; this approach may assist future healthcare workers in delivering services that significantly benefit their communities. However, studies with larger sample sizes and a more balanced distribution of students are warranted, as they will provide a better understanding of how students choose a variety of medical specialties.
